# The characteristics of homicide perpetrators in a medium secure forensic hospital: A study from dundrum hospital

**DOI:** 10.1192/j.eurpsy.2021.1003

**Published:** 2021-08-13

**Authors:** N. Basrak, H. Kennedy, M. Davoren

**Affiliations:** 1 National Forensic Mental Health Service, Central Mental Hospital Dundrum, Dundrum, Ireland; 2 The Dundrum Centre For Forensic Excellence, Trinity College Dublin, Dublin, Ireland

**Keywords:** Homicide, forensic psychiatry

## Abstract

**Introduction:**

The majority of homicides in society are not associated with mental illness, however there is an established association between homicide and schizophrenia. Homicide perpetrated by mentally disordered offenders is a leading reason for admission to secure forensic psychiatric hospitals.

**Objectives:**

To investigate the clinical characteristics of those with a history of completed homicide in the CMH Dundrum.

**Methods:**

This study was a cross sectional study of a cohort of patients in the Central Mental Hospital who had completed homicide (n=63).

**Results:**

A total of 136 patients were included, 46.3% (n=63) of whom had committed homicide. Mean age of homicide perpetrators at admission was 34.6 years old (median 33.4, s.d. = 9.72). The most common diagnosis was schizophrenia (n=40, 63.5%). 73.0% (n=46) had a history of substance misuse. 36.5% (n=23) had a diagnosis of a personality disorder, including traits only. The most common victim type was a family member (n=32, 50.8%). Patients with a history of homicide had better scores on dynamic risk of violence (F=8.553, p=0.004), programme completion (F=8.258, p=0.005) and recovery (F=3.666, p=0.058) compared to non-homicide offenders, however they also had significantly longer mean length of stay, 12.7 years v 7.5 years (F=9.634,p=0.002).

**Conclusions:**

Homicide perpetrators with a mental illness constitute a significant portion of the forensic mental health population and a high number of these offences were against family members. A history of homicide among forensic in-patients is associated with a longer length of stay which has implications for service development into the future.

